# In Vitro Azole Susceptibility of *Malassezia globosa* Isolates from Healthy Controls, Atopic Dermatitis, and Pityriasis Versicolor Across Anatomical Sites

**DOI:** 10.3390/jof12100773

**Published:** 2026-10-15

**Authors:** Yu Yu, Xiaochun Liu, Yuan Zhou, Huan Mei, Weida Liu, Xiaodong She, Xu Yao

**Affiliations:** 1 Department of Allergy and Rheumatology, Jiangsu Provincial Key Laboratory of Dermatology, Hospital for Skin Diseases, Institute of Dermatology, Chinese Academy of Medical Sciences & Peking Union Medical College, Nanjing 210042, China; 2 Department of Medical Mycology, Jiangsu Provincial Key Laboratory of Dermatology, Hospital for Skin Diseases, Institute of Dermatology, Chinese Academy of Medical Sciences & Peking Union Medical College, Nanjing 210042, China

**Keywords:** *Malassezia globosa*, antifungal susceptibility, azoles, atopic dermatitis, pityriasis versicolor, minimum inhibitory concentration

## Abstract

*Malassezia globosa* is a major member of the human skin mycobiota and is associated with several cutaneous disorders, yet it remains unclear whether its in vitro azole susceptibility varies with disease status or anatomical site. We evaluated 55 *M. globosa* isolates obtained from five healthy controls, five patients with atopic dermatitis (AD), and five patients with pityriasis versicolor (PV). Isolates were collected from the forehead, posterior neck, and antecubital fossa in all participants, with additional lesional isolates from AD and PV. Minimum inhibitory concentrations (MICs) were determined for fluconazole, ketoconazole, itraconazole, posaconazole, voriconazole, and isavuconazole. Fluconazole showed the broadest MIC range (0.5–64 μg/mL), whereas posaconazole and voriconazole showed lower ranges (0.00375–0.5 μg/mL). Mixed-effects analyses of the three shared non-lesional sites detected no significant disease-group, site, or disease-by-site effects for any drug (all *p* > 0.05). Repeated-measures analyses within AD and PV likewise found no significant overall site effect when lesional skin was included. These exploratory data indicate substantial inter-isolate heterogeneity but no consistent association between *M. globosa* azole susceptibility and clinical group, anatomical site, or lesional status. Larger standardized and strain-resolved studies are warranted.

## 1. Introduction

The human skin harbors a complex microbial ecosystem in which fungi contribute to cutaneous homeostasis and immune regulation [1,2,3]. Among these fungi, species of the genus *Malassezia* are the predominant commensal yeasts on healthy human skin, particularly in sebaceous areas where they can account for the majority of the cutaneous mycobiota [4]. Owing to their lipid dependence, *Malassezia* species preferentially colonize lipid-rich sites such as the scalp, face, neck, and upper trunk. Although generally commensal, alterations in *Malassezia* abundance or composition have been associated with pityriasis versicolor (PV), seborrheic dermatitis, *Malassezia* folliculitis, psoriasis, and atopic dermatitis (AD) [5,6].

Among the human-associated species, *Malassezia globosa*, *M. restricta*, and *M. sympodialis* are frequently detected on normal skin [3,7]. Their distribution varies according to anatomical site, host characteristics, environmental factors, and disease status. *M. globosa* is one of the most prevalent species on healthy skin and is also considered an important etiological species in PV [4,6,8]. Increasing evidence further suggests that *M. globosa* may contribute to AD through interactions with the epidermal barrier and host immune responses [9]. However, whether *M. globosa* isolates recovered from different disease backgrounds or skin sites exhibit distinct biological phenotypes remains insufficiently understood.

Azole antifungal agents are widely used for the treatment of *Malassezia*-associated dermatoses [6]. Previous studies have shown substantial interspecies variation in antifungal susceptibility, with ketoconazole and itraconazole generally showing greater in vitro activity, whereas fluconazole often exhibits higher minimum inhibitory concentrations (MICs). Nevertheless, susceptibility testing of *Malassezia* remains technically challenging because of its lipid requirements and the lack of universally standardized methods. Moreover, most available studies have focused on differences among *Malassezia* species rather than variation within a single species [10,11,12]. Consequently, relatively little is known about whether the clinical origin of *M. globosa*, including disease status and anatomical site, is associated with altered antifungal susceptibility.

This question is relevant because healthy skin, AD, and PV represent distinct cutaneous microenvironments with differences in lipid composition, barrier integrity, local inflammation, and microbial community structure [13]. Such ecological differences may influence fungal physiology and potentially modify antifungal responses. Accurate species identification is therefore essential when evaluating susceptibility patterns. Internal transcribed spacer (ITS) sequencing provides reliable molecular discrimination of closely related *Malassezia* species and reduces the risk of phenotypic misidentification [4,14].

Given the distinct cutaneous microenvironments represented by healthy skin, AD, and PV, we hypothesized that *M. globosa* isolates recovered from different clinical backgrounds and anatomical sites might exhibit distinct azole susceptibility profiles. Specifically, we sought to determine whether MICs differed according to clinical group, anatomical site, or lesional status. To address this question while minimizing interspecies variability, we restricted the analysis to molecularly confirmed *M. globosa* isolates and evaluated their susceptibility to six azole antifungal agents. This study was therefore designed to characterize intraspecies variation in azole susceptibility and to explore whether clinical or anatomical origin is associated with reproducible differences in the antifungal phenotype of *M. globosa*.

## 2. Materials and Methods

### 2.1. Study Participants

Participants were recruited and classified into three groups: healthy controls (HCs), patients with atopic dermatitis (AD), and patients with pityriasis versicolor (PV). A total of 15 participants were included, comprising five healthy controls, five patients with AD, and five patients with PV. The present study was designed as an exploratory investigation, and no formal a priori power calculation was performed. The number of participants was determined based on the availability of eligible participants during the study period and the ability to obtain *M. globosa* isolates from the predefined anatomical sites required by the study design. The diagnosis of AD and PV was established by dermatologists based on clinical evaluation. Healthy controls had no clinically apparent inflammatory or infectious skin disease at the time of sampling.

For healthy controls, samples were obtained from three predefined anatomical sites: the forehead, posterior neck, and antecubital fossa. Patients with AD and PV were sampled from the same three anatomical sites, with an additional sample collected from lesional skin.

The study was conducted in accordance with the Declaration of Helsinki and was approved by the Hospital for Skin Diseases, the Institute of Dermatology, the Chinese Academy of Medical Sciences, and Peking Union Medical College (CAMS & PUMC) (approval no. 2025-079; date of approval: 10 December 2025). Written informed consent was obtained from all participants prior to sample collection.

### 2.2. Skin Sampling and Isolation of Malassezia

Skin samples were collected using sterile cotton swabs. A standardized area of approximately 2 × 2 cm^2^ was sampled at each anatomical site. The skin surface was rubbed continuously with a sterile cotton swab for 40 s to ensure adequate collection of cutaneous microorganisms.

Immediately after sampling, each swab was evenly streaked onto modified Dixon (mDixon) agar supplemented with 0.05% chloramphenicol and 0.05% cycloheximide for the primary isolation of *Malassezia*. The inoculated plates were incubated for 10 days at 35 °C for *Malassezia* growth.

After primary culture, an isolated yeast colony was randomly selected from each culture-positive plate and subcultured on fresh mDixon agar to obtain a pure expanded culture. The purified isolates were subsequently used for molecular identification and antifungal susceptibility testing.

### 2.3. DNA Extraction and Molecular Identification of Malassezia globosa

Genomic DNA was extracted from purified *Malassezia* cultures using MasterPure™ Yeast DNA Purification Kit (LGC Biosearch Technologies, Middleton, WI, USA; Cat. No. MPY80200), according to the manufacturer’s instructions.

Species-level identification was performed by sequencing the internal transcribed spacer (ITS) region of fungal ribosomal DNA. The ITS region was amplified by polymerase chain reaction (PCR) using the universal fungal primers ITS1 (5′-TCCGTAGGTGAACCTGCGG-3′) and ITS4 (5′-TCCTCCGCTTATTGATATGC-3′), as previously described by White et al. [15]. PCR products were purified and subjected to Sanger sequencing by General Biol (Anhui) Co., Ltd., Chuzhou, Anhui, China.

The resulting sequences were assembled and compared with reference sequences deposited in NCBI GenBank using BLAST + 2.17.0. The nucleotide sequences have been deposited in GenBank under accession numbers QB030504–QB030558. Isolates showing sequence identity consistent with *Malassezia globosa* were included in the subsequent antifungal susceptibility analysis.

For the present study, 55 molecularly identified *M. globosa* isolates were included, comprising 15 isolates from healthy controls, 20 from patients with AD, and 20 from patients with PV.

### 2.4. Antifungal Susceptibility Testing

Antifungal susceptibility testing was performed using an optimized colorimetric broth microdilution method adapted from Leong et al. with minor modifications [10]. Six azole antifungal agents were tested: fluconazole (FLC), ketoconazole (KTZ), itraconazole (ITZ), posaconazole (POS), voriconazole (VOR), and isavuconazole (ISA). All antifungal compounds were purchased from MedChemExpress (MCE, Monmouth Junction, NJ, USA).

Stock solutions of the six azole antifungal agents were prepared in dimethyl sulfoxide (DMSO) based on the general procedures described in CLSI M27-Ed4 [16]. The stock concentrations were 12,800 μg/mL for FLC, 1600 μg/mL for KTZ, 800 μg/mL for ITZ, 1000 μg/mL for POS, 1000 μg/mL for VOR, and 4000 μg/mL for ISA. The stock solutions were subsequently diluted in OptiMAL broth to prepare the required two-fold concentrated working solutions. Because the stock solutions were substantially more concentrated than the final test concentrations, the carryover of DMSO into the assay was limited; based on the stock and final test concentrations, the highest theoretical final DMSO concentration was approximately 0.5%.

Susceptibility testing was performed in OptiMAL broth, an RPMI 1640-based medium optimized for the growth of lipid-dependent *Malassezia* species. The medium contained 0.165 M MOPS, 0.2 mg/mL sodium bicarbonate, 2% glucose, 0.5% glycerol, 0.5% Tween 60, 2% oleic acid, 0.1% esculin, and 12.5 μg/mL resazurin, with a final pH of approximately 6.26.

Two-fold serial dilutions of each antifungal agent were prepared as two-fold concentrated working solutions. The final concentration ranges tested were as follows: FLC, 0.5–64 μg/mL; KTZ, 0.06–8 μg/mL; ITZ, 0.015–2 μg/mL; POS, 0.00375–0.5 μg/mL; VOR, 0.00375–0.5 μg/mL; and ISA, 0.015–2 μg/mL. In each well of a sterile 96-well microtiter plate, 100 μL of the standardized yeast suspension was combined with 100 μL of the corresponding two-fold concentrated antifungal solution, resulting in a final volume of 200 μL per well and a final inoculum density of 5.0 × 10^3^ to 5.0 × 10^4^ CFU/mL. The sterility control consisted of 200 μL of drug-free OptiMAL broth, whereas the growth control consisted of 100 μL of drug-free OptiMAL broth and 100 μL of the standardized yeast suspension. Microplates were incubated at 35 °C until adequate color development was observed in the drug-free growth control. Resazurin served as a metabolic growth indicator, with viable fungal growth producing a color change from blue to pink. MIC endpoints were determined by visual colorimetric assessment. For all azole antifungal agents, the minimum inhibitory concentration (MIC) was defined as the lowest antifungal concentration producing at least 50% inhibition of fungal growth relative to the drug-free growth control.

*Candida parapsilosis* ATCC 22019 was included as a quality control strain to assess the accuracy of antifungal dilutions and the reproducibility of the broth microdilution assay, consistent with the quality-control approach used in the optimized Leong method.

Each *M. globosa* isolate was tested in at least duplicate experiments. When replicate MIC values differed by more than two two-fold dilution steps, the assay was repeated. MIC values were expressed in μg/mL.

### 2.5. Statistical Analysis

MIC values were log_2_-transformed before statistical analysis to account for the two-fold dilution structure of the broth microdilution assay and to improve visualization.

To investigate whether antifungal susceptibility differed according to clinical group and anatomical site, the primary analysis was restricted to the three non-lesional anatomical sites shared by all study groups: the forehead (F), posterior neck (N), and antecubital fossa (A).

For each antifungal agent, a mixed-effects model was fitted with clinical group (HCs, AD, and PV), anatomical site, and the clinical group × anatomical site interaction as fixed effects. Participant identity was included as a random effect to account for repeated measurements obtained from multiple anatomical sites within the same individual. Anatomical site was treated as a repeated-measures factor. The Geisser–Greenhouse correction was applied without assuming sphericity.

Because lesional samples were available only from patients with AD and PV, lesion-associated analyses were performed separately within each disease group. MIC values obtained from the forehead, posterior neck, antecubital fossa, and lesional skin were compared using repeated-measures one-way analysis of variance (RM one-way ANOVA) with the Geisser–Greenhouse correction. Dunnett’s multiple-comparisons test was subsequently used to compare isolates obtained from lesional skin with those from each non-lesional anatomical site.

Heatmaps were generated using log_2_-transformed MIC values to visualize antifungal susceptibility profiles across isolates. A two-sided *p* value < 0.05 was considered statistically significant. Statistical analyses were performed using GraphPad Prism version 10.1.0 (GraphPad Software, Boston, MA, USA).

## 3. Results

### 3.1. Participant Characteristics and Isolate Distribution

Fifteen participants were included, with five participants in each clinical group. The median ages were 28 years in HCs, 24 years in AD, and 38 years in PV. Each participant contributed isolates from the forehead, posterior neck, and antecubital fossa; patients with AD and PV additionally contributed one lesional isolate. A total of 55 *M. globosa* isolates were therefore analyzed: 15 from HCs, 20 from AD, and 20 from PV (Table 1).

### 3.2. Distribution of MICs Across the Six Azoles

**Figure 1:**
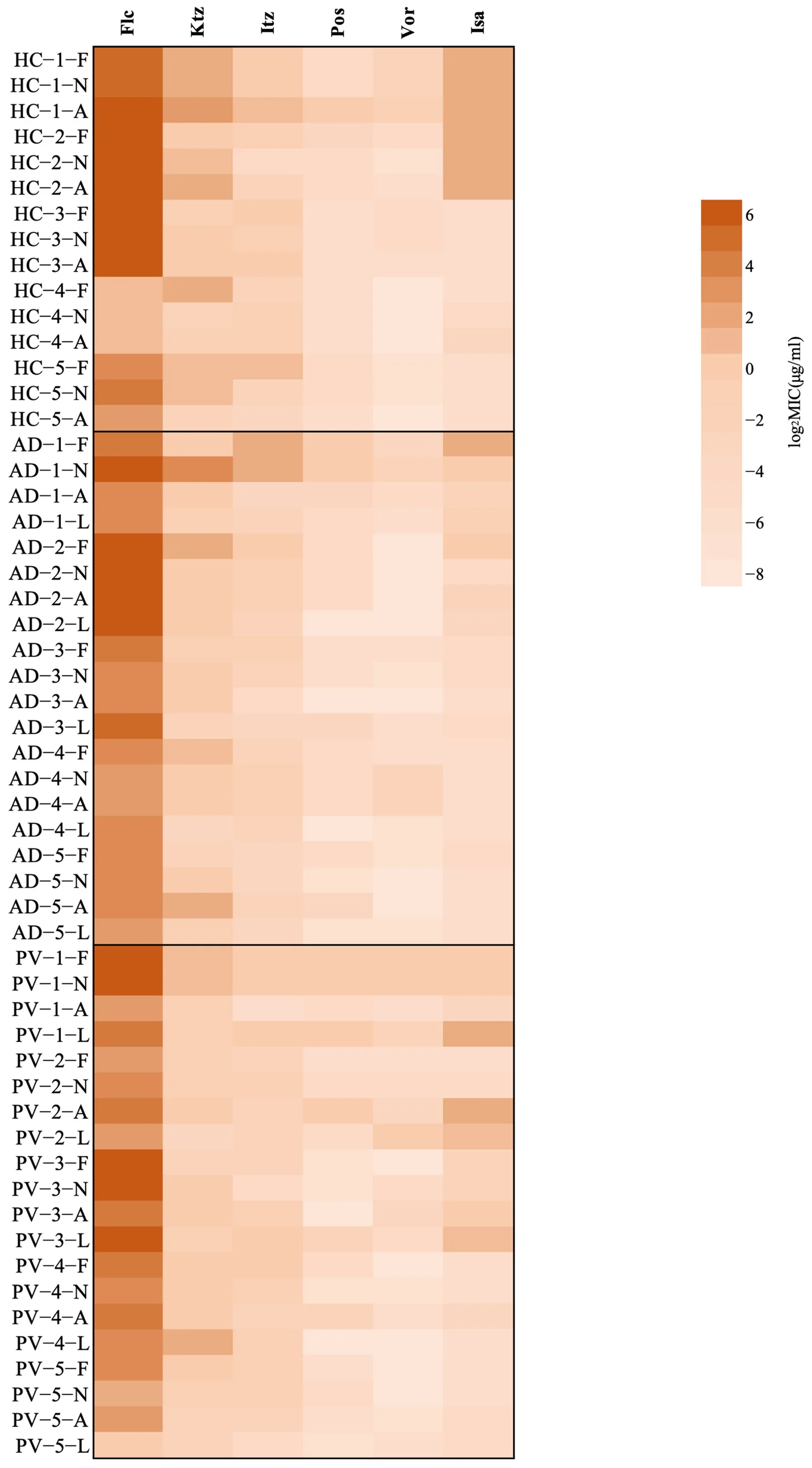
Heatmap of antifungal susceptibility profiles of *Malassezia globosa* isolates. Minimum inhibitory concentrations (MICs) of fluconazole (FLC), ketoconazole (KTZ), itraconazole (ITZ), posaconazole (POS), voriconazole (VOR), and isavuconazole (ISA) were determined for 55 *M. globosa* isolates obtained from healthy controls (HCs), patients with atopic dermatitis (AD), and patients with pityriasis versicolor (PV). MIC values were log_2_-transformed for visualization. Rows represent individual isolates and columns represent antifungal agents; isolates are arranged according to clinical group, participant, and anatomical sampling site. F, forehead; N, posterior neck; A, antecubital fossa; L, lesional skin.

Considerable isolate-to-isolate variability was observed across the six antifungal agents (Table S1 and Figure 1). The observed MIC ranges were 0.5–64 μg/mL for FLC, 0.06–8 μg/mL for KTZ, 0.015–2 μg/mL for ITZ, 0.00375–0.5 μg/mL for POS, 0.00375–0.5 μg/mL for VOR, and 0.015–2 μg/mL for ISA. FLC showed the broadest and highest MIC distribution, whereas POS and VOR showed lower MIC ranges. The heatmap of log_2_-transformed MICs illustrated substantial heterogeneity among individual isolates without an obvious pattern defined by clinical group or sampling site.

### 3.3. Effects of Disease Group and Anatomical Site

**Figure 2:**
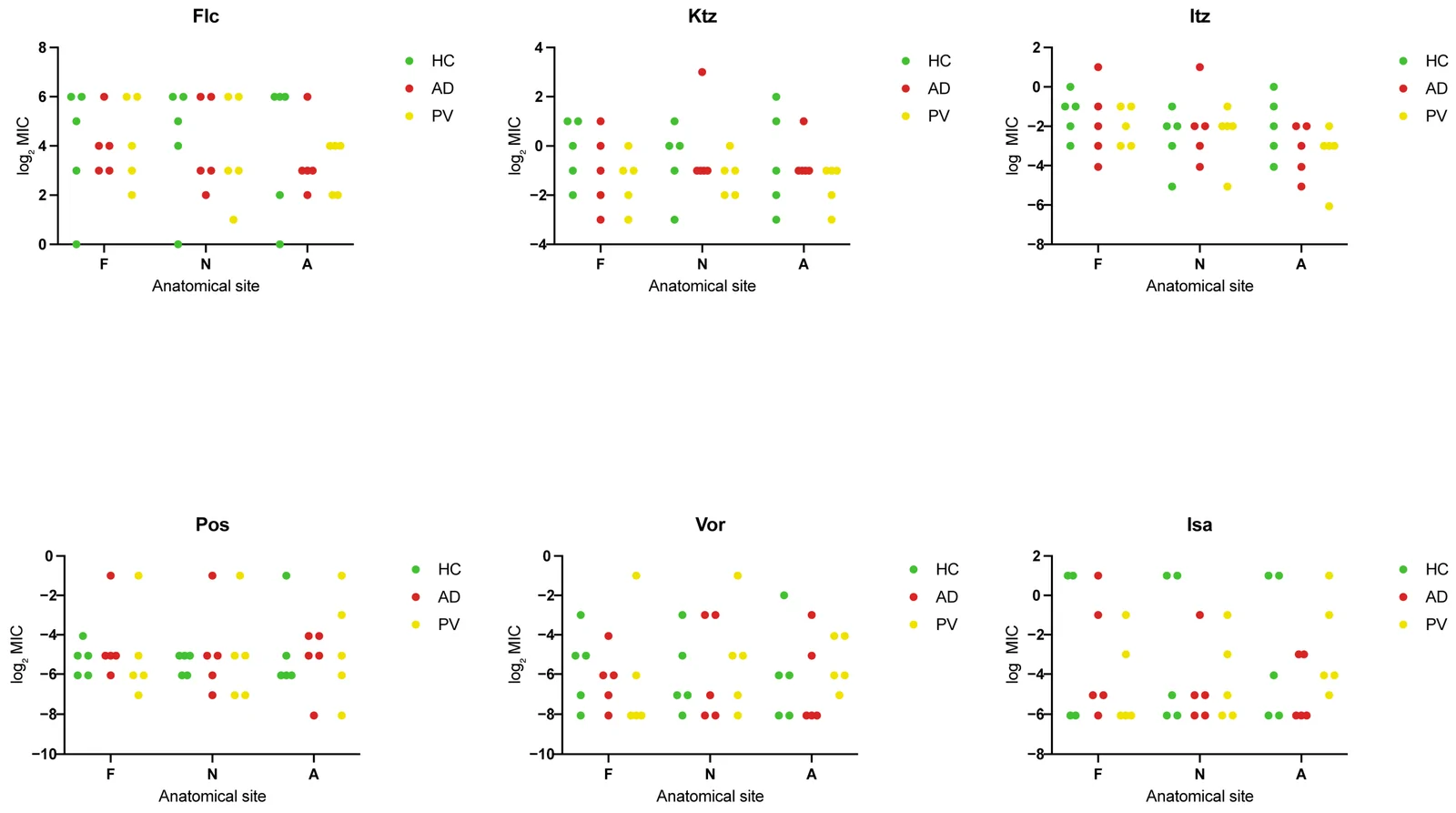
Effects of clinical group and anatomical site on the antifungal susceptibility of *Malassezia globosa* isolates. MIC values were log_2_-transformed. Each dot represents an isolate obtained from one participant at the forehead (F), posterior neck (N), or antecubital fossa (A). Differences were assessed using mixed-effects models with disease group and anatomical site as fixed effects, participant as a random effect, and anatomical site as a repeated-measures factor. No significant disease-group, anatomical-site, or disease group × site effects were observed for any of the six antifungal agents (all *p* > 0.05).

The primary mixed-effects analysis included only F, N, and A, which were sampled in all three clinical groups. No significant main effect of disease group was detected for FLC (*p* = 0.9566), KTZ (*p* = 0.1934), ITZ (*p* = 0.6316), POS (*p* = 0.9375), VOR (*p* = 0.9183), or ISA (*p* = 0.7766). Anatomical site likewise showed no significant effect for any drug (FLC, *p* = 0.2807; KTZ, *p* = 0.8481; ITZ, *p* = 0.1337; POS, *p* = 0.8148; VOR, *p* = 0.6791; ISA, *p* = 0.4886). No disease group × site interaction reached statistical significance (all *p* > 0.10) (Table 2; Figure 2).

Although the fixed effects of disease group, site, and their interaction were non-significant, the repeated-measures output indicated significant participant-level clustering for FLC, POS, VOR, and ISA, suggesting that susceptibility profiles may show inter-individual heterogeneity not captured by clinical group or anatomical site. This observation was interpreted descriptively because of the small number of participants.

### 3.4. Lesional and Non-Lesional Sites Within AD and PV

Because healthy participants had no lesional skin sample, lesion-associated analyses were performed separately within AD and PV. In AD, repeated-measures one-way ANOVA across F, N, A, and L detected no significant overall site effect for FLC (*p* = 0.5670), KTZ (*p* = 0.1511), ITZ (*p* = 0.2202), POS (*p* = 0.2794), VOR (*p* = 0.5331), or ISA (*p* = 0.1093). Likewise, no significant overall site effect was detected in PV for FLC (*p* = 0.3707), KTZ (*p* = 0.6673), ITZ (*p* = 0.5379), POS (*p* = 0.9365), VOR (*p* = 0.5219), or ISA (*p* = 0.2027). Subsequent Dunnett-adjusted comparisons between lesional skin and each non-lesional anatomical site were also non-significant for all six azoles in both the AD and PV groups (all adjusted *p* > 0.05).

**Figure 3:**
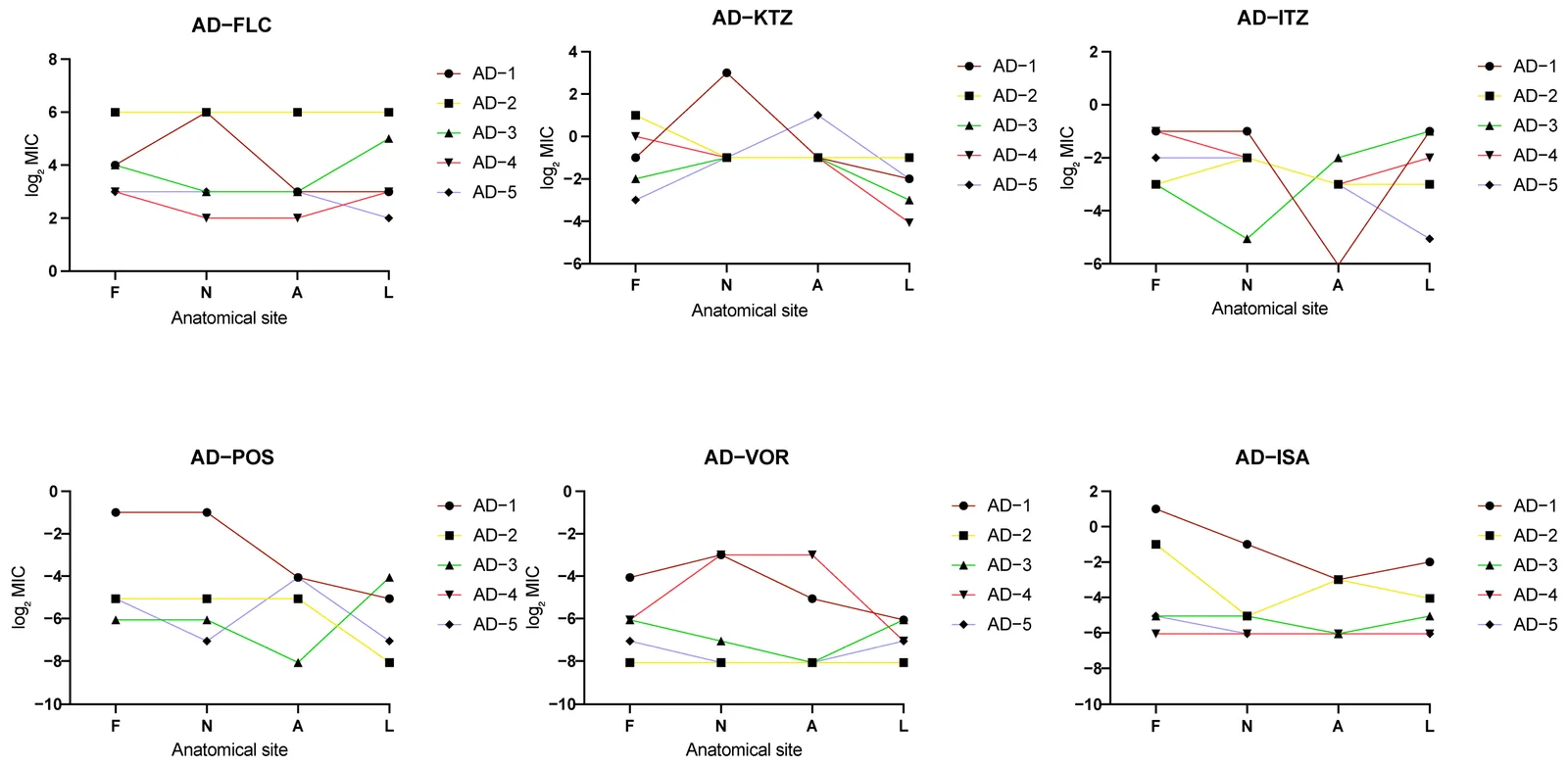
Antifungal susceptibility of *Malassezia globosa* isolates from different anatomical sites in patients with atopic dermatitis. MIC values were log_2_-transformed. Connected points represent isolates obtained from the same patient across the forehead (F), posterior neck (N), antecubital fossa (A), and lesional skin (L). Differences were assessed using repeated-measures one-way ANOVA with the Geisser–Greenhouse correction, followed by Dunnett-adjusted comparisons of L with each non-lesional site.

**Figure 4:**
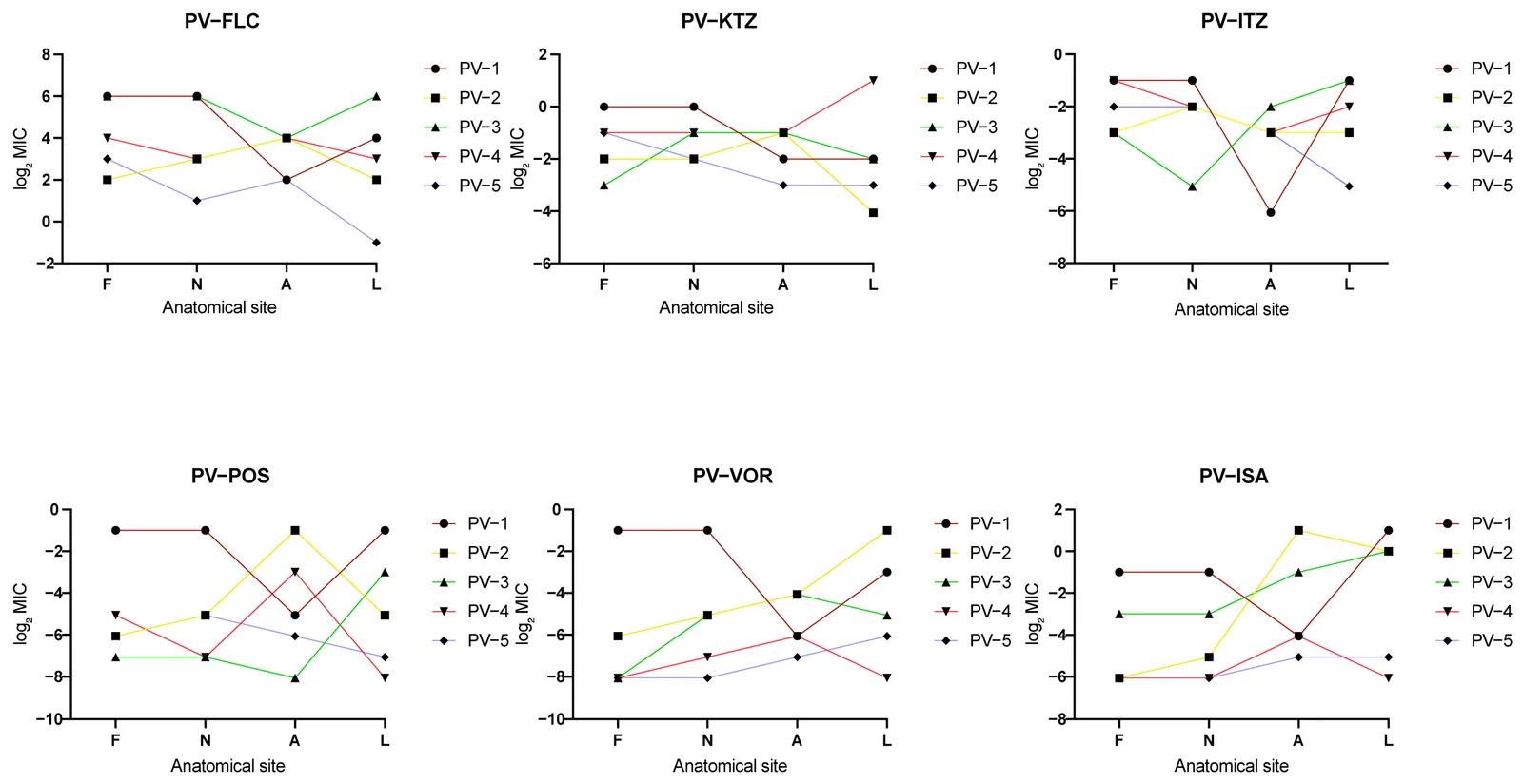
Antifungal susceptibility of *Malassezia globosa* isolates from different anatomical sites in patients with pityriasis versicolor. MIC values were log_2_-transformed. Connected points represent isolates obtained from the same patient across the forehead (F), posterior neck (N), antecubital fossa (A), and lesional skin (L). Differences were assessed using repeated-measures one-way ANOVA with the Geisser–Greenhouse correction, followed by Dunnett-adjusted comparisons of L with each non-lesional site.

Each connected line in Figure 3 and Figure 4 represents an individual participant and illustrates the within-participant variation in MIC values across different anatomical sites. Although individual trajectories varied, no consistent increase or decrease in MICs was observed from non-lesional to lesional skin in either the AD or PV group.

These findings provide no evidence, in this small paired dataset, for a consistent lesion-associated shift in *M. globosa* azole susceptibility (Figure 3 and Figure 4).

## 4. Discussion

This study evaluated six azole antifungal agents against 55 molecularly identified *M. globosa* isolates obtained through repeated multisite sampling of healthy controls and patients with AD or PV. Three main observations emerged. First, MICs varied substantially among individual isolates, with FLC showing the broadest and highest distribution and POS and VOR showing lower MIC ranges. Second, when repeated sampling within participants was accounted for, no significant association was detected between MICs and clinical group, anatomical site, or the interaction between these factors. Third, inclusion of lesional skin in within-disease repeated-measures analyses did not identify a significant overall site effect in either AD or PV. Taken together, these data suggest that the marked phenotypic heterogeneity observed among *M. globosa* isolates was not organized in a simple disease- or site-specific pattern in this exploratory cohort.

The broad FLC distribution is compatible with prior *Malassezia* susceptibility studies [10,11,12,17,18,19,20,21]. Modified broth microdilution studies have repeatedly reported higher or more variable MICs for FLC than for several other azoles, although absolute values vary according to species and testing methodology [22]. Notably, Li et al. evaluated 50 *M. globosa* isolates recovered from patients with *Malassezia* folliculitis or PV and from healthy subjects and reported MICs below 1 μg/mL for ITZ, POS, VOR, and KTZ [23]. Earlier modified broth microdilution studies including *M. globosa* similarly demonstrated marked species- and drug-dependent variation in azole susceptibility [24,25]. A 2022 *Journal of Fungi* study of 58 *Malassezia* isolates from PV lesions and healthy skin also found substantial between-drug and between-species differences [21], while a 2026 study of Brazilian PV and seborrheic dermatitis isolates again showed lower MICs for ITZ and KTZ than for several comparator agents [26]. These observations are broadly consistent with the relatively low POS and VOR MICs observed in the present study. Our study extends previous work by focusing exclusively on *M. globosa* and by accounting for the dependency among multiple isolates obtained from the same participant.

No significant disease-group effect was detected for any of the six azoles. This finding suggests that, within the limits of the present sample, isolates recovered from AD or PV did not show a consistent shift in azole susceptibility relative to isolates from healthy controls [27]. This is biologically plausible because the role of *Malassezia* differs across these conditions. In PV, *M. globosa* can participate directly in superficial fungal disease, whereas in AD the importance of *Malassezia* is more commonly linked to altered mycobiome structure, barrier dysfunction, and host immune responses. Importantly, the absence of a disease-associated MIC shift does not necessarily imply an absence of disease-associated strain-level variation. Sugita et al. demonstrated substantial intraspecific genetic diversity in *M. globosa* and reported differences in IGS1 genotype distributions between isolates colonizing patients with AD and healthy individuals [28]. Thus, disease-associated ecological selection may affect genetic or functional traits without necessarily producing a consistent shift in conventional azole MICs.

Anatomical site also did not significantly influence MICs across the forehead, posterior neck, and antecubital fossa. Skin fungal communities are strongly shaped by anatomical topography. Findley et al. demonstrated that although *Malassezia* dominates many core and sebaceous skin sites, fungal community composition varies substantially among anatomical locations [29]. These site-associated patterns are thought to reflect local differences in sebum, temperature, humidity, and other physiological characteristics. Nevertheless, our findings suggest that such topographic ecological variation does not necessarily translate into a reproducible site-specific shift in azole susceptibility within *M. globosa*. Similarly, the separate AD and PV analyses provided no evidence of an overall difference among lesional and non-lesional sampling sites. These negative findings should be interpreted as an absence of statistically detectable effects rather than proof of biological equivalence, because each disease group contained only five participants.

The evidence of participant-level clustering for several agents is potentially informative. Previous molecular typing studies demonstrate substantial intraspecific diversity within *M. globosa*. Hadrich et al. identified 24 multilocus genotypes among 44 *M. globosa* isolates, illustrating considerable strain-level diversity within this species [30]. Multiple isolates from a given individual may therefore be genetically related or may have been shaped by a shared host microenvironment and antifungal-exposure history. Thus, between-host variability may contribute to susceptibility heterogeneity more strongly than the broad diagnostic categories examined here.

Interpretation of *Malassezia* MICs remains constrained by the lack of validated species-specific clinical breakpoints. In addition, different laboratories have used modified CLSI- or EUCAST-based methods with different lipid-supplemented media, inoculum densities, incubation conditions, and MIC endpoints [10,12,25,31]. Rojas et al. proposed tentative epidemiological cutoff values for several *Malassezia* species, including *M. globosa* [32]. However, epidemiological cutoff values distinguish putative wild-type from non-wild-type populations and should not be interpreted as clinical susceptibility breakpoints. Therefore, isolates with relatively high MICs in the present study should not be classified as clinically resistant by extrapolating breakpoints established for *Candida* spp. Their main value is as phenotypic observations that may help identify unusual isolates for future genomic and mechanistic study.

This study has several limitations. Most importantly, only five participants were included in each clinical group, so the effective number of independent hosts was small despite analysis of 55 isolates. The study was therefore underpowered to detect modest disease- or site-associated effects and cannot establish equivalence between groups. The cross-sectional design does not address temporal stability of susceptibility. In addition, *Malassezia* susceptibility testing is intrinsically method-dependent, making direct comparison of absolute MIC values across studies difficult [10,12,25,31]. Clinical antifungal exposure and treatment outcomes were not incorporated into the current analysis, and no molecular investigation of high-MIC isolates was performed. These limitations should be considered when interpreting the absence of significant group-level effects.

Nevertheless, the study has several strengths. All isolates belonged to a single molecularly identified species, sampling was standardized across three anatomical sites in all clinical groups, and the statistical analysis respected the repeated-measures structure created by multiple isolates from the same participant. Lesional skin was analyzed separately rather than being treated as a conventional fourth anatomical site in the between-group model. The inclusion of six azoles, including POS, VOR, and ISA, provides a broad descriptive susceptibility dataset for *M. globosa*. The inclusion of ISA is particularly relevant because susceptibility data for this newer triazole remain limited for *Malassezia*. Rathie et al. evaluated ISA against *M. restricta*, *M. slooffiae*, and *M. sympodialis* and demonstrated measurable in vitro activity, but *M. globosa* was not included [11]. This adds descriptive value to the ISA data generated in the present investigation. Larger multicenter studies combining harmonized susceptibility testing with strain-resolved genomics, longitudinal sampling, antifungal-exposure histories, and clinical outcomes are warranted.

## 5. Conclusions

*M. globosa* isolates from healthy controls and patients with AD or PV showed substantial heterogeneity in azole MICs, with FLC displaying the broadest distribution. However, no statistically significant disease-group, anatomical-site, disease-by-site, or overall lesional-site effect was detected for any of the six azoles examined. The observed participant-level heterogeneity suggests that host-specific or strain-level factors may be more important determinants of MIC variation than broad clinical diagnosis or sampling site. Because *Malassezia*-specific clinical breakpoints are not established, these results should be interpreted as phenotypic susceptibility distributions rather than clinical resistance classifications. Larger standardized studies integrating genomic characterization are needed to define the biological and clinical relevance of high-MIC *M. globosa* isolates.

## Figures and Tables

**Table 1:** Characteristics of participants and distribution of *Malassezia globosa* isolates.

Characteristics	HCs (*n* = 5)	AD (*n* = 5)	PV (*n* = 5)
Participant characteristics			
Age median	28	24	38
Male/female	2/3	3/2	2/3
Distribution of *M. globosa* isolates			
Forehead isolates, *n*	5	5	5
Posterior neck isolates, *n*	5	5	5
Antecubital fossa isolates, *n*	5	5	5
Lesional isolates, *n*	-	5	5
Total isolates, *n*	15	20	20

Abbreviations: HCs, healthy controls; AD, atopic dermatitis; PV, pityriasis versicolor.

**Table 2:** Mixed-effects analysis of disease group and anatomical site effects on antifungal susceptibility of *Malassezia globosa* isolates.

Drug	Disease Group, *p*	Site Group, *p*	Disease × Site *p*
FLC	0.9566	0.2807	0.8408
KTZ	0.1934	0.8481	0.9041
ITZ	0.6316	0.1337	0.6737
POS	0.9375	0.8148	0.8820
VOR	0.9183	0.6791	0.7480
ISA	0.7766	0.4886	0.1041

*p* values were obtained from models fitted separately for each antifungal agent using log_2_-transformed MIC values. Disease group and anatomical site were included as fixed effects, participant as a random effect, and anatomical site as a repeated-measures factor.

## Data Availability

The nucleotide sequences generated in this study are available in GenBank under accession numbers QB030504–QB030558. All MIC data supporting the findings of this study are provided within the article. Requests for materials should be addressed to yypumc@163.com.

## Supplementary Materials

The following supporting information can be downloaded at https://www.mdpi.com/article/10.3390/jof12100773/s1, Table S1: Individual minimum inhibitory concentration values of 55 *Malassezia globosa* isolates.

## Abbreviations

The following abbreviations are used in this manuscript:

HCs	Healthy controls
AD	Atopic dermatitis
PV	Pityriasis versicolor
FLC	Fluconazole
KTZ	Ketoconazole
ITZ	Itraconazole
VOR	Voriconazole
POS	Posaconazole
ISA	Isavuconazole
