# Supplementary material for: In Vitro Azole Susceptibility of Malassezia globosa Isolates from Healthy Controls, Atopic Dermatitis, and Pityriasis Versicolor Across Anatomical Sites
Source: J Fungi (Basel). 2026 Oct 15;12(10):773. doi: 10.3390/jof12100773 (PMC13705733; doi:10.3390/jof12100773)
Supplement: Supplementary file 1 [file jof-12-00773-s001.zip › jof-4618602-supplementary.pdf]

**Table S1.** Individual minimum inhibitory concentration values of 55 *Malassezia globosa* isolates.

| | MIC(μg/mL) | | | | | |
|---|---|---|---|---|---|---|
| | FLC | KTZ | ITZ | POS | VOR | ISA |
| HC-1-F | 32 | 2 | 0.5 | 0.03 | 0.125 | 2 |
| HC-1-N | 32 | 2 | 0.5 | 0.03 | 0.125 | 2 |
| HC-1-A | 64 | 4 | 1 | 0.5 | 0.25 | 2 |
| HC-2-F | 64 | 0.5 | 0.25 | 0.06 | 0.03 | 2 |
| HC-2-N | 64 | 1 | 0.03 | 0.03 | 0.0075 | 2 |
| HC-2-A | 64 | 2 | 0.125 | 0.03 | 0.015 | 2 |
| HC-3-F | 64 | 0.25 | 0.5 | 0.015 | 0.03 | 0.015 |
| HC-3-N | 64 | 0.5 | 0.25 | 0.015 | 0.03 | 0.015 |
| HC-3-A | 64 | 0.5 | 0.5 | 0.015 | 0.015 | 0.015 |
| HC-4-F | 1 | 2 | 0.125 | 0.015 | 0.00375 | 0.015 |
| HC-4-N | 1 | 0.125 | 0.25 | 0.015 | 0.00375 | 0.03 |
| HC-4-A | 1 | 0.25 | 0.25 | 0.015 | 0.00375 | 0.06 |
| HC-5-F | 8 | 1 | 1 | 0.03 | 0.0075 | 0.015 |
| HC-5-N | 16 | 1 | 0.125 | 0.03 | 0.0075 | 0.015 |
| HC-5-A | 4 | 0.125 | 0.06 | 0.015 | 0.00375 | 0.015 |
| AD-1-F | 16 | 0.5 | 2 | 0.5 | 0.06 | 2 |
| AD-1-N | 64 | 8 | 2 | 0.5 | 0.125 | 0.5 |
| AD-1-A | 8 | 0.5 | 0.06 | 0.06 | 0.03 | 0.125 |
| AD-1-L | 8 | 0.25 | 0.125 | 0.03 | 0.015 | 0.25 |
| AD-2-F | 64 | 2 | 0.5 | 0.03 | 0.00375 | 0.5 |
| AD-2-N | 64 | 0.5 | 0.25 | 0.03 | 0.00375 | 0.03 |
| AD-2-A | 64 | 0.5 | 0.25 | 0.03 | 0.00375 | 0.125 |
| AD-2-L | 64 | 0.5 | 0.125 | 0.00375 | 0.00375 | 0.06 |
| AD-3-F | 16 | 0.25 | 0.25 | 0.015 | 0.015 | 0.03 |
| AD-3-N | 8 | 0.5 | 0.125 | 0.015 | 0.0075 | 0.03 |
| AD-3-A | 8 | 0.5 | 0.03 | 0.00375 | 0.00375 | 0.015 |
| AD-3-L | 32 | 0.125 | 0.06 | 0.06 | 0.015 | 0.03 |
| AD-4-F | 8 | 1 | 0.125 | 0.03 | 0.015 | 0.015 |
| AD-4-N | 4 | 0.5 | 0.25 | 0.03 | 0.125 | 0.015 |
| AD-4-A | 4 | 0.5 | 0.25 | 0.03 | 0.125 | 0.015 |
| AD-4-L | 8 | 0.06 | 0.125 | 0.00375 | 0.0075 | 0.015 |
| AD-5-F | 8 | 0.125 | 0.06 | 0.03 | 0.0075 | 0.03 |
| AD-5-N | 8 | 0.5 | 0.06 | 0.0075 | 0.00375 | 0.015 |
| AD-5-A | 8 | 2 | 0.125 | 0.06 | 0.00375 | 0.015 |
| AD-5-L | 4 | 0.25 | 0.06 | 0.0075 | 0.0075 | 0.015 |
| PV-1-F | 64 | 1 | 0.5 | 0.5 | 0.5 | 0.5 |
| PV-1-N | 64 | 1 | 0.5 | 0.5 | 0.5 | 0.5 |
| PV-1-A | 4 | 0.25 | 0.015 | 0.03 | 0.015 | 0.06 |
| PV-1-L | 16 | 0.25 | 0.5 | 0.5 | 0.125 | 2 |
| PV-2-F | 4 | 0.25 | 0.125 | 0.015 | 0.015 | 0.015 |
| PV-2-N | 8 | 0.25 | 0.25 | 0.03 | 0.03 | 0.03 |
| PV-2-A | 16 | 0.5 | 0.125 | 0.5 | 0.06 | 2 |
| PV-2-L | 4 | 0.06 | 0.125 | 0.03 | 0.5 | 1 |
| PV-3-F | 64 | 0.125 | 0.125 | 0.0075 | 0.00375 | 0.125 |
| PV-3-N | 64 | 0.5 | 0.03 | 0.0075 | 0.03 | 0.125 |

| | | | | | | |
|---|---|---|---|---|---|---|
| PV-3-A | 16 | 0.5 | 0.25 | 0.00375 | 0.06 | 0.5 |
| PV-3-L | 64 | 0.25 | 0.5 | 0.125 | 0.03 | 1 |
| PV-4-F | 16 | 0.5 | 0.5 | 0.03 | 0.00375 | 0.015 |
| PV-4-N | 8 | 0.5 | 0.25 | 0.0075 | 0.0075 | 0.015 |
| PV-4-A | 16 | 0.5 | 0.125 | 0.125 | 0.015 | 0.06 |
| PV-4-L | 8 | 2 | 0.25 | 0.00375 | 0.00375 | 0.015 |
| PV-5-F | 8 | 0.5 | 0.25 | 0.015 | 0.00375 | 0.015 |
| PV-5-N | 2 | 0.25 | 0.25 | 0.03 | 0.00375 | 0.015 |
| PV-5-A | 4 | 0.125 | 0.125 | 0.015 | 0.0075 | 0.03 |
| PV-5-L | 0.5 | 0.125 | 0.03 | 0.0075 | 0.015 | 0.03 |

Abbreviations: F, forehead; N, posterior neck; A, antecubital fossa; L, lesional skin.
